# Stalling of Eukaryotic Translesion DNA Polymerases at DNA-Protein Cross-Links

**DOI:** 10.3390/genes13020166

**Published:** 2022-01-18

**Authors:** Anna V. Yudkina, Evgeniy S. Shilkin, Alena V. Makarova, Dmitry O. Zharkov

**Affiliations:** 1 Siberian Branch of the Russian Academy of Sciences Institute of Chemical Biology and Fundamental Medicine, 8 Lavrentieva Ave., 630090 Novosibirsk, Russia; 2 Institute of Molecular Genetics, National Research Center «Kurchatov Institute», 2 Kurchatov sq., 123182 Moscow, Russia; shilkinevgeniy.chem@gmail.com (E.S.S.); amakarova-img@yandex.ru (A.V.M.); 3 Department of Natural Sciences, Novosibirsk State University, 2 Pirogova St., 630090 Novosibirsk, Russia

**Keywords:** DNA–protein cross-link, DNA polymerases, DNA replication, translesion synthesis

## Abstract

DNA-protein cross-links (DPCs) are extremely bulky adducts that interfere with replication. In human cells, they are processed by SPRTN, a protease activated by DNA polymerases stuck at DPCs. We have recently proposed the mechanism of the interaction of DNA polymerases with DPCs, involving a clash of protein surfaces followed by the distortion of the cross-linked protein. Here, we used a model DPC, located in the single-stranded template, the template strand of double-stranded DNA, or the displaced strand, to study the eukaryotic translesion DNA polymerases ζ (POLζ), ι (POLι) and η (POLη). POLι demonstrated poor synthesis on the DPC-containing substrates. POLζ and POLη paused at sites dictated by the footprints of the polymerase and the cross-linked protein. Beyond that, POLζ was able to elongate the primer to the cross-link site when a DPC was in the template. Surprisingly, POLη was not only able to reach the cross-link site but also incorporated 1–2 nucleotides past it, which makes POLη the most efficient DNA polymerase on DPC-containing substrates. However, a DPC in the displaced strand was an insurmountable obstacle for all polymerases, which stalled several nucleotides before the cross-link site. Overall, the behavior of translesion polymerases agrees with the model of protein clash and distortion described above.

## 1. Introduction

DNA is always associated with a variety of proteins. However, this tight association increases the risk of haphazard covalent attachment of proteins to DNA with the formation of DNA-protein cross-links (DPCs). DPCs can be produced by a variety of proteins, many of which form stable nucleoprotein complexes with DNA or participate in DNA metabolism [1]. DPCs are quite ubiquitous lesions: estimates based on different approaches put their amount at 0.5–70 per 10^7^ bases, and this number rises with age [1,2,3].

Compared with other DNA lesions, DPCs are extremely bulky. Therefore, their presence in the genome could strongly interfere with vital cellular processes, such as replication [4], transcription [5,6], chromatin remodeling [7,8], and DNA topology manipulation [9,10]. Moreover, DPCs may cause DNA fragmentation and disrupt methylation patterns, leading, eventually, to cancer or cell death. On the other hand, specific DPCs can shield more deleterious DNA lesions from unwanted reactions to protect them until properly repaired [11,12,13].

DPCs could be generated by a variety of endogenous or environmental sources. Cross-linking can be induced by metabolic or xenobiotic aldehydes [14,15,16], chemotherapeutical drugs [17], ionizing and UV radiation, and oxidative stress [1,3,18]. Moreover, DPCs can be classified as non-enzymatic DPCs, which are generated by the non-specific covalent trapping of proteins by genotoxic agents, and enzymatic DPCs, which are produced by errors of DNA-processing enzymes [19]. Since many enzymes form reversible covalent intermediates with DNA during catalysis, these intermediates may be erroneously diverted to dead-end DPCs under certain circumstances. The best-known examples of such enzymes are DNA topoisomerases [9,20,21], DNA polymerases [22,23,24], DNA methyltransferases [4], DNA glycosylases [23,25,26], and poly(ADP-ribose) polymerases [27]. As cross-linking occurs accidentally, DPCs are highly heterogeneous with respect to the nature of the cross-linked proteins, their size, DNA sequence context, chemistry of the covalent linkage, etc. Often, a DPC is accompanied with DNA breaks, which could be located at different positions relative to the cross-link point [19,28,29].

So far, DPCs remain the most poorly studied types of DNA damage. Several breakthrough works in recent years have revealed the mechanism of initiation of their repair and established a link between the repair and replication stalling by DPCs [30]. Despite the DPCs’ heterogeneity and multiple pathways of recognition and processing, their repair almost universally starts from degradation of the protein part by specific highly conserved proteases or by the ubiquitin-dependent proteasome, followed by translesion synthesis or the recruitment of excision repair enzymes [30].

Importantly, the activity of SPRTN, one of the main proteases involved in DPC repair, has been shown to be triggered by DNA polymerases stalled near DPCs [30,31,32,33,34,35]. However, the impact of a DPC on a replisome depends on a complex of the DPC’s properties, and little is known about the interactions of DNA polymerases with DPCs. Several works demonstrated that model DPCs completely block replicative and repair DNA polymerases in vitro [17,36,37,38,39]. However, the polymerases show widely varying ability to elongate the primer in the presence of a DPC, with some stopping almost immediately after the collision with the surface of the cross-linked protein and others extending almost to the point of the cross-link, which apparently requires major deformation of the DPC’s protein part. This could possibly facilitate DPC recognition by downstream repair proteases.

The presence of unrepaired lesions of DNA in the S phase of the cell cycle may be tolerated through the process known as translesion DNA synthesis (TLS). TLS involves a set of specific DNA polymerases that have a wide and flexible active site with no substrate conformational selection and lack proofreading, which helps them to bypass the lesion at the expense of low synthesis accuracy. Most of these polymerases belong to the structural family Y, which, in human cells, encompasses DNA polymerases ι, κ, η, and Rev1. Moreover, to extend aberrant primer termini after lesion bypasses, another TLS polymerase is recruited, namely DNA polymerase ζ of the structural family B, which uses alternative DNA alignment to extend the 3′-mispaired primer [40,41]. However, the behavior of human TLS polymerases encountering extremely bulky DPCs have been addressed before only for DNA polymerase κ, which stalled several nucleotides before the cross-linking site [38].

**Figure 1:**
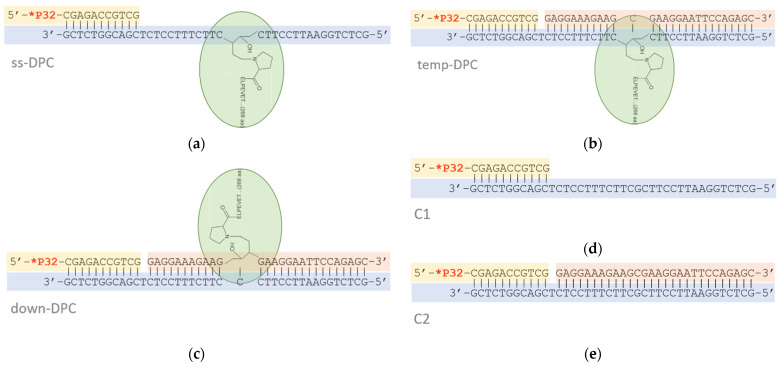
Schemes of the model substrates. (**a**) ss-DPC, (**b**) temp-DPC, (**c**) down-DPC, (**d**) control C1, (**e**), control C2. Fpg protein (green) is cross-linked to DNA through the reduced Schiff base with Pro1. The primer is ^32^P-labeled.

In this work, we have investigated whether DNA polymerases ι (POLι), η (POLη), and ζ (POLζ), which are involved in TLS in eukaryotic cells, are able to bypass a DPC or reach the cross-link site when the DPC is located in the template strand of single-stranded (ss-DPC) or double-stranded DNA (temp-DPC), or in the non-template displaced strand of a DNA duplex (down-DPC) (Figure 1). Although none of them was able to fully bypass the DPC, POLζ and POLη supported primer extension up to or even beyond the cross-link site in the DNA template, suggestive of their ability to distort the cross-linked protein. On the contrary, a DPC in the displaced strand represented an insurmountable obstacle for the TLS DNA polymerases.

## 2. Materials and Methods

### 2.1. Oligonucleotides and Enzymes

Yeast DNA polymerase η (yPOLη), human DNA polymerase η (hPOLη), human DNA polymerase ι (hPOLι), four-subunit yeast DNA polymerase ζ (yPOLζ), and *E*. *coli* formamidopyrimidine-DNA glycosylase (Fpg) were overexpressed and purified essentially as described [42,43,44]. Oligonucleotides (Table 1) were synthesized in-house from commercially available phosphoramidites (Glen Research, Sterling, VA, USA) and purified by reverse-phase HPLC on a PRP-1 C18 column (Hamilton, Reno, NV, USA). If necessary, oligonucleotides were 5′-labeled using γ[^32^P]-ATP and phage T4 polynucleotide kinase (SibEnzyme, Novosibirsk, Russia), according to the manufacturer’s protocol. The sequences of the oligonucleotides used in this study are shown in Table 1 and the positions of the DNA-protein cross-link sites are indicated.

### 2.2. Preparation of DNA-Protein Cross-Links

Model DPCs between the oligonucleotides and Fpg were prepared by NaBH_4_ cross-linking and purified, as previously described [38,45]. The DPC-containing substrates were annealed to the ^32^P-labeled primer for 30 min at room temperature.

### 2.3. Primer Extension Reactions

Primer extension reactions (20 μL) contained 10 nM DPC substrate (see above), DNA polymerase (100 nM for hPOLη, yPOLη, and hPOLι or 40 nM for yPOLζ), the dNTP mixture of 50 μM of each, 30 mM HEPES (pH 7.4), 5% glycerol, 0.1 mg/mL of bovine serum albumin, and 10 mM MgCl_2_ or 0.5 mM MnCl_2_. The reaction was allowed to proceed at 37 °C for 2, 5, or 30 min. At these times, aliquots were withdrawn and an equal volume of a loading solution (95% formamide, 20 mM Na-EDTA, and 0.1% bromophenol blue) was added, followed by 2 min of heating at 95 °C. The reaction products were resolved by 21% denaturing polyacrylamide gel electrophoresis and visualized by phosphorimaging (Typhoon FLA 9500, GE Healthcare, Chicago, IL, USA). The lengths of the extension products were determined from comparisons with the mobility markers (Table 1) and partial extension ladders.

## 3. Results

### 3.1. Substrate Design

The schemes of the model substrates are shown in Figure 1. The substrates contained a DPC in the template strand of single-stranded DNA (ss-DPC), in the template strand of double-stranded DNA (temp-DPC) and in the displaced strand of double-stranded DNA (down-DPC). As controls, we used primer–template (C1) or primer–displaced strand–template (C2) constructs without the damaged base that were not subjected to the cross-linking procedure.

### 3.2. Inefficient Synthesis by POLι on DPC-Containing Substrates

**Figure 2:**
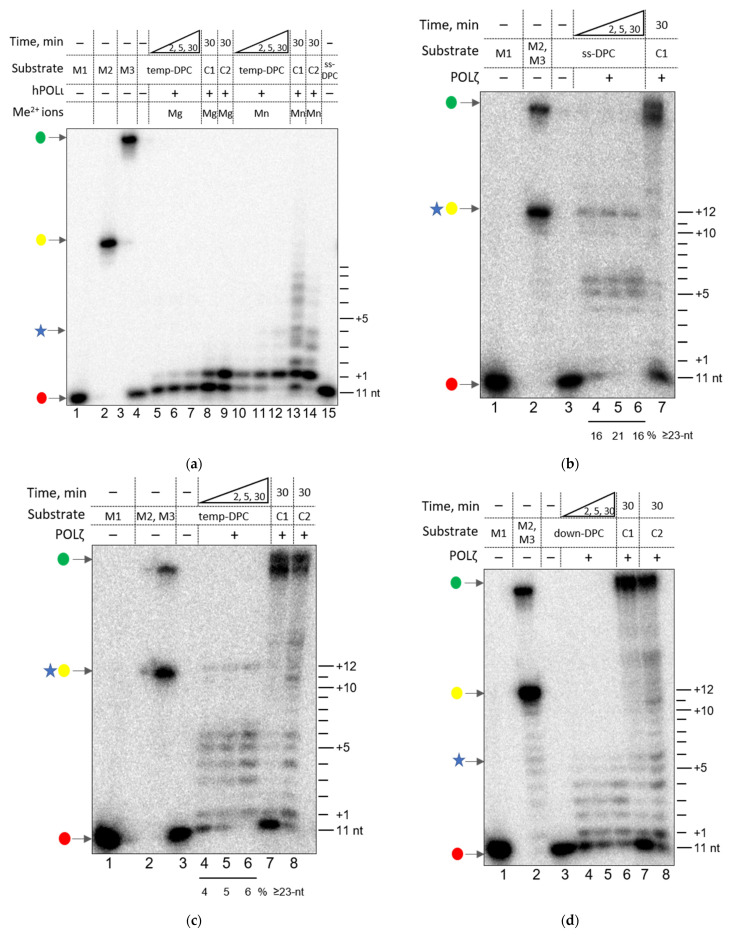
Primer elongation by hPOLι (**a**) and yPOLζ (**b**–**d**) encountering a DNA protein cross-link. For yPOLζ, panel (**b**) shows the ss-DPC substrate, panel (**c**) shows the temp-DPC substrate, and panel (**d**) shows the down-DPC substrate. The red dots mark the primers, the yellow dots correspond to the synthesis until the cross-link site, and the green dots indicate the full-sized products. The star shows the maximal primer extension on each DPC-containing substrate by each DNA polymerase. Panel (**a**): lanes 1–3, size markers 11 nt (M1), 23 nt (M2), and 40 nt long (M3); lanes 4 and 15, temp-DPC (lane 4) or ss-DPC substrate (lane 15), no DNA polymerase; lanes 5–7: primer extension for 2, 5, and 30 min, respectively, with Mg^2+^; lanes 10–12, same with Mn^2+^; lanes 8–9, primer extension (30 min) on the undamaged primer–template (C1, lane 8) or primer–displaced strand–template substrate (C2, lane 9); lane 13–14, same with Mn^2+^. Panels (**b**–**d**): lanes 1–2, size markers M1, M2 and M3; lane 3, ss-DPC (**b**), temp-DPC (**c**) or down-DPC substrates (**d**), no DNA polymerase; lanes 4–6: primer extension for 2, 5, and 30 min, respectively; lanes 7–8, primer extension (30 min) on the undamaged primer–template (C1, lane 7) or primer–displaced strand–template substrate (C2, lane 8).

POLι is a human family Y DNA polymerase, and its function in vivo has not been elucidated yet. To investigate the ability of hPOLι to approach a DPC, we performed primer extension reactions on the model DPC-containing substrates in the presence of dNTPs and Mg^2+^ or Mn^2+^ ions (Figure 2a). hPOLι is a low-processivity DNA polymerase, typically incorporating 1–3 nucleotides per association [46]. However, it is more active on gapped DNA substrates incorporating 7–10 nucleotides and possesses limited strand displacement activity [47,48].

In the presence of Mg^2+^, hPOLι stopped synthesis after the incorporation of a single nucleotide (Figure 2a, lanes 8–9). This is likely a consequence of the “T-stop”, a unique feature of hPOLι that frequently misincorporates dGMP opposite to T and poorly extends such a mismatched terminus [49,50]. Thus, we used Mn^2+^ instead, which noticeably stimulated the activity; under these conditions, hPOLι incorporated 7–10 nucleotides into the primer annealed to the undamaged template (C1) (Figure 2a, lane 13) and 3–5 nucleotides if the substrate also contained a displaced strand (C2) (Figure 2a, lane 14). However, even in the presence of Mn^2+^ ions, hPOLι activity was insufficient to estimate the polymerase behavior in the presence of DPCs, mostly leading to primer elongation by one nucleotide (Figure 2a, lanes 10–12). The model DPC-containing substrates were designed to provide unobstructed DNA polymerase substrate binding and the incorporation of 2–3 nucleotides before the contact of the polymerase with the cross-linked protein’s surface. As a distributive DNA polymerase, hPOLι releases the substrate after the incorporation of a few nucleotides, even with Mn^2+^, and reassociation of the enzyme for further synthesis in the vicinity of the cross-linked protein could be complicated. Additionally, it has previously been demonstrated that a DPC may stabilize the displaced strand and impede its displacement by DNA polymerases [38]. Clearly, hPOLι is inefficient in DNA synthesis near a DPC.

### 3.3. POLζ Is Able to Reach the Cross-Link Site

POLζ is a processive family B DNA polymerase that participates in translesion DNA synthesis, usually as an “extender” polymerase [41,51]. However, POLζ is also moderately proficient in synthesis over bulky obstacles of different origins, such as *cis-syn* thymine dimers, (6-4) photoproducts, and 1,2-intrastrand cisplatin cross-links [45,52,53], pointing to a potential additional role of POLζ as yet another translesion DNA polymerase. Moreover, the REV1-POLζ complex is required for TLS across DNA–peptide adducts [35,54], suggesting a possible interaction of POLζ with DPCs.

We performed primer extension experiments with yeast POLζ on the DPC-containing substrates in the presence of Mg^2+^ ions (Figure 2b–d). During the synthesis on the undamaged substrate (C1), yPOLζ efficiently extended the primer: we observed a full-sized product without any noticeable accumulation of shorter products, which generally correspond to DNA polymerase pausing or partially stalling (Figure 2b–d, lane 7). However, during the synthesis on the substrate containing a DPC in the template of the single-stranded substrate (ss-DPC), yPOLζ demonstrated two pause points at the +5 and +6 positions after the 3′ end of the primer (Figure 2b, lanes 4–6). These pause points correspond to 4–5 nucleotides before to the cross-link site position. Despite the pause in the synthesis, yPOLζ was able to elongate the primer to the cross-link site directly (23-mer product), which means yPOLζ was able to reach the cross-link site, despite the presence of a bulky protein adduct (Figure 2b, lanes 4–6). These data are fully consistent with the “kiss-and-push” model from our previous work [38]. Like other members of family B DNA polymerases, yPOLζ was able to reach the cross-link site by distorting (“pushing”) the covalently attached protein, whereas the pause 4–5 nucleotides before the cross-link site appears when the protein surfaces of the DPC and the elongating DNA polymerase first come into contact. Notably, the same pause points were demonstrated by most of the studied DNA-polymerases in our previous work [38].

POLζ is known to possess strand displacement activity [55,56]. Interestingly, on the control substrate, yPOLζ showed some pause points, which may reflect local difficulties in strand displacement. The yPOLζ-catalyzed synthesis on the substrate containing a DPC in the template strand of double-stranded DNA (temp-DPC) was similar to the synthesis on the ss-DPC substrate (Figure 2c), strongly terminating at the same positions +5…+6. However, the presence of the displaced strand apparently complicated “pushing” of the DPC by yPOLζ: a band corresponding to the 23-nt product was of lower intensity, whereas the pause points were more pronounced (Figure 2c, lanes 4–6). Additional pause points appeared at the +3 and +4 positions (Figure 2c, lanes 4–6), which are likely explained by the additive effect of the complementary strand displacement and the cross-linked protein.

The synthesis on the substrates containing a DPC in the displaced strand (down-DPC) does not involve translesion synthesis. However, in our previous work [38] it was shown that DNA polymerases were not able to fully extend the primer while a DPC was in the displaced strand and, moreover, did it even less efficiently than with a DPC in the template strand. yPOLζ demonstrated the same behavior (Figure 2d). The longest elongation products on this substrate corresponded to the incorporation of 5–6 nucleotides (Figure 2d, lanes 4–6).

### 3.4. POLη Able to Reach the Cross-Link Site and Beyond

POLη is an archetypal eukaryotic translesion DNA polymerase of family Y. The best-known POLη role is the protection against UV lesions by accurately copying opposite cyclobutane pyrimidine dimers [57,58]. Human (hPOLη) and yeast POLη (yPOLη) have low fidelity on the undamaged substrate but are able to bypass several bulky adducts, such as cisplatin G-G interstrand cross-links [59], acetylaminofluorene-dG [59], and thymine *cis-syn* dimers [60], with relatively high fidelity.

**Figure 3:**
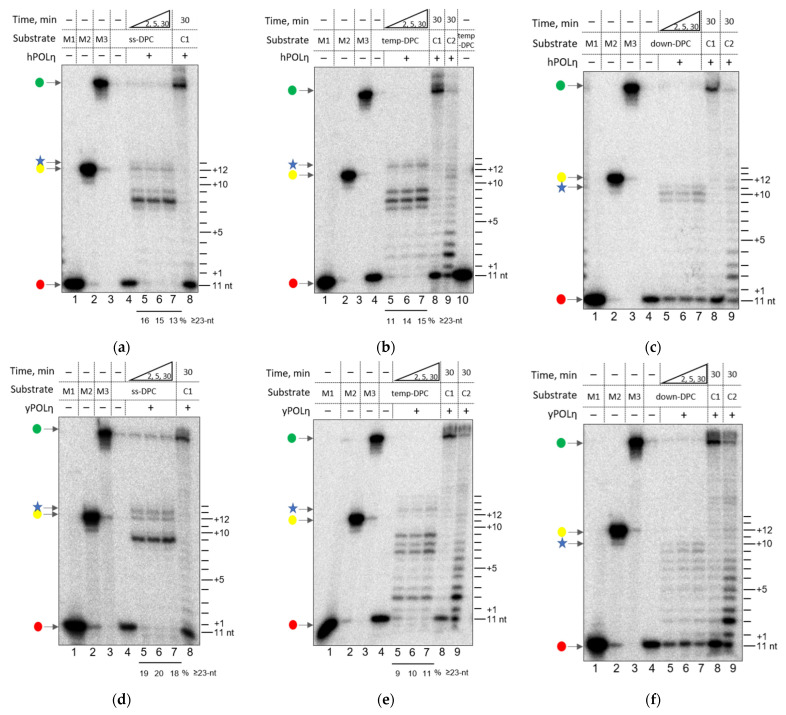
Primer elongation by hPOLη (**a**–**c**) and yPOLη (**d**–**f**) encountering a DNA protein cross-link. Panels (**a**,**d**), ss-DPC substrate; panels (**b**,**e**), temp-DPC substrate; panels (**c**,**f**), down-DPC substrate. The red dots mark the primers, the yellow dots correspond to the synthesis until the cross-link site, and the green dots indicate the full-sized products. Lanes 1–3, size markers 11 nt (M1), 23 nt (M2), and 40 nt long (M3); lane 4 (also lane 10 in Panel (**b**)), the respective substrate in the absence of DNA polymerase; lanes 5–7, primer extension for 2, 5, and 30 min, respectively; lanes 8–9, primer extension (30 min) on the undamaged primer–template (C1, lane 8) or primer–displaced strand–template substrate (C2, lane 9).

We performed a similar set of primer extension experiments with hPOLη on the DPC-containing substrates in the presence of Mg^2+^ ions (Figure 3a–c). In our previous study, the family Y DNA polymerase POLκ stalled before the cross-link site on the ssDNA containing a DPC, while *Sulfolobus solfataricus* Dpo4 had a very limited ability to reach the cross-link site [38]. Surprisingly, hPOLη clearly demonstrated the ability to reach the cross-link site (Figure 3a, lanes 5–7, 23-mer primer elongation product). Generally, the synthesis on the substrates containing a DPC in the single-stranded template was similar to the synthesis by family B DNA polymerases [38]. Significant synthesis pause points were also observed 3–4 nucleotides before the cross-link site (Figure 3a, lanes 5–7). Unexpectedly, we consistently observed some low-intensity image density above the 23-mer product, possibly indicating insertion of a dNMP beyond the cross-link site (Figure 3a, lanes 5–7).

hPOLη primer elongation on the temp-DPC substrates confirmed these results. It was even more evident that hPOLη can incorporate 1–2 nucleotides past the cross-link site (Figure 3b, lanes 5–7). hPOLη also demonstrated a pause 3–4 nucleotides before a DPC (Figure 3b, lanes 5–7). The ability of hPOLη to elongate the primer past a DPC was quite unexpected. In our model system, DNA polymerases encounter a cross-linked protein surface soon after the start of the synthesis (the “kiss” stage of the interaction of DNA polymerases with DPCs [38]). This event leads to pause points several nucleotides before the cross-link site, with the exact location depending on the size and footprint of the polymerase and the protein obstacle. Further synthesis is due to the ability of a DNA polymerase to “push” the cross-linked protein by distorting its structure. This allows further primer elongation; however, the push also distorts the DNA polymerase itself. hPOLη stopped synthesis two nucleotides past the cross-link site, which likely means a full distortion of the cross-linked protein, yet the polymerase still keeps the ability to incorporate dNMPs. Alternatively, hPOLη might bypass a DPC by using a DNA lesion “skipping” mechanism on a template with a repetitive C or expand the 3′-G following repetitive primer dislocation and slippage.

To corroborate these results, we also investigated the activity of yPOLη using the same model substrates. yPOLη demonstrated a very similar behavior to hPOLη on the substrate containing DPC in the template strand of single-stranded (Figure 3d) or double-stranded DNA (Figure 3e). The stop points of yPOLη past the cross-link site were even more pronounced than those of hPOLη (Figure 3d,e, lanes 5–7) The synthesis on the substrates containing a DPC in the displaced strand was also quite efficient: both human and yeast POLη stopped 2–3 nucleotides before the cross-link site, which is, again, the best result compared to the other studied DNA polymerases (Figure 3c,f, lanes 5–7). Therefore, POLη seems to be the most efficient DNA polymerase in its ability to carry out DNA synthesis in the presence of DPCs, either in the template strand or in the displaced strand.

## 4. Discussion

DNA–protein cross-link repair is tightly coupled with replication and begins with proteolysis of the protein part to a small peptide by the proteasome or one of the dedicated proteases [31,35,54]. It is assumed that the main protease involved in the replication-coupled degradation of DPCs, SPRTN, processes a cross-link to a short peptide, and these small peptides should be bypassed by translesion DNA polymerases [35] since large peptide adducts will presumably block DNA synthesis. However, the mechanism of coordination between replication and protease engagement is not fully understood at present. There are many tightly bound proteins and slow-turnover enzymes that process DNA and remain bound to it for a long time [61,62,63]. For cellular systems, a protein cross-linked to DNA may be barely distinguishable from a protein tightly bound to DNA in a non-covalent manner. Therefore, the mechanism of true DPC recognition by proteases is crucial for DPC repair.

One way to trigger DPC removal is stalling the replication-associated CMG helicase at the protein adduct in the leading strand [30]. This CMG stalling leads to DPC ubiquitylation and proteolysis. Surprisingly, ubiquitylation is not essential for SPRTN-dependent DPC degradation [35]. This SPRTN property is conditioned by its targeting to DPCs by DNA polymerases [31,32,33]. SPRTN requires a DNA polymerase to approach within a few nucleotides of a DPC if CMG is not stalled, e.g., if a DPC is in the leading strand but passable through the CMG’s central canal, if a DPC is in the leading strand behind the replication fork, if a DPC is in the lagging strand, or during gap filling synthesis [30,31,34,35]. DNA polymerase inhibition 16 nucleotides upstream of the DPC does not lead to SPRTN recruiting [35]. Thus, the ability of DNA polymerases to proceed with the synthesis in the vicinity of a DPC and to interact with a DPC directly is of great interest.

The specific DNA polymerases involved in the SPRTN targeting remain ambiguous. On one hand, a variety of biologically significant events ending in DPC formation may occur throughout the cell cycle and expose DPCs to various replicative, translesion, or repair DNA polymerases. On the other hand, the specific properties of individual DNA polymerases, such as their structural features or the ability to proceed with translesion synthesis, may result in different products of synthesis in the vicinity of a DPC. Moreover, because of the extremely bulky nature of DPCs, primer elongation near a DPC likely involves the interaction of the DNA polymerase and the cross-linked protein, with the possible distortion of one or both proteins. It is unclear whether this protein globule deformation is irreversible, which may be highly undesirable if a replicative DNA polymerase such as POLδ or POLε approaches a DPC to trigger SPRTN. Alternatively, partial unfolding of the protein part of a DPC could expose the buried peptides, thereby triggering its recognition by proteases or tagging it for proteasomal degradation. Investigations of the interactions of individual DNA polymerases with model DPCs could reveal some of these aspects. In the current work, we have investigated the ability of translesion DNA polymerases to deal with DPCs.

In our previous study [38] we suggested a mechanism of interaction of DNA polymerases with DPCs in terms of protein surface contacts, which could account for the observed heterogeneity of polymerase pause or termination sites on such substrates. We termed it the “kiss-and-push” model. The “kiss” stage corresponds to the meeting of the surfaces of the DNA polymerase and the cross-linked protein. Most DNA polymerases show a pause of the synthesis at this stage, with its position almost exactly predictable from the proteins’ footprints, as observed in their crystal structures. The next stage, the “push”, corresponds to the ability of some (but not all) DNA polymerases to distort the cross-linked protein globule to read through, in some cases, up to the very site of cross-linking. The data obtained in the present work are fully consistent with this model.

Since DPCs may be formed with a variety of proteins, the interactions of DNA polymerases with DPCs are likely non-specific and guided only by the configuration of the interacting protein surfaces and the polymerase properties. Interestingly but not unexpectedly, DNA polymerases belonging to the same structural family tend to show similar properties when interacting with the model DPCs. This is especially evident with family B DNA polymerases (phage RB69 and T4 polymerases in [38] and yPOLζ in the present work), which are able to extend the primer to the cross-link site. This ability is probably due to the high processivity of family B polymerases, which allows them to distort the cross-linked protein significantly without DNA polymerase dissociation, because the reassociation of DNA polymerase with DNA in the immediate vicinity of a DPC is unlikely.

**Figure 4:**
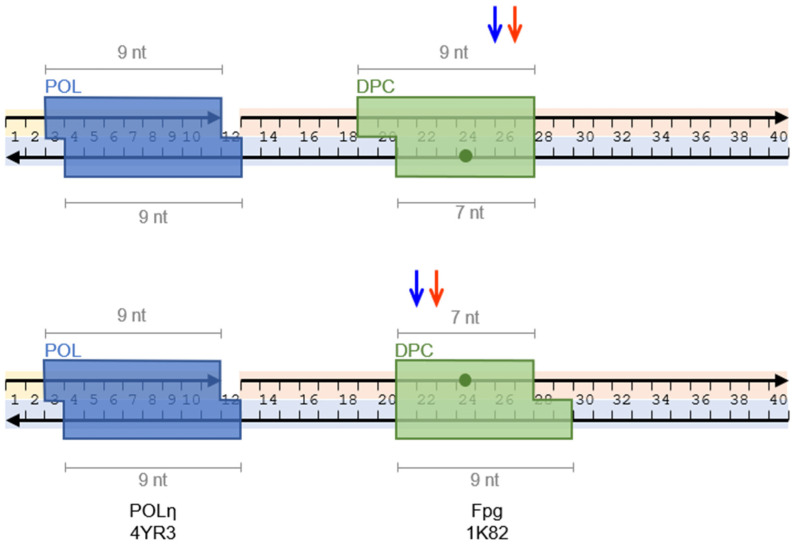
An arrangement of hPOLη and cross-linked Fpg on DNA with the sizes of the proteins estimated from the structural data. PDB IDs are 1K82 for the structure of Fpg [42] and 4YR3 for the structure of hPOLη [78]. Arrowheads mark the 3′ termini of the oligonucleotides. The green dots indicate the sites of cross-linking. The colored arrows indicate the termination positions of hPOLη while Fpg is cross-linked with the template strand (top panel) or the displaced strand (bottom panel). The blue arrows mark the positions of the last incorporated dNMP, the red arrows show the corresponding positions of the estimated front sides of hPOLη.

In family Y, which evolved for translesion synthesis, we earlier observed that *S. solfataricus* Dpo4 has a very limited ability to reach the cross-link site if a DPC was in a single-stranded template, while hPOLκ stopped ~4 nt before the cross-link [38]. While family Y hPOLι, due to its low processivity, did not approach a DPC closely, hPOLη showed an ability to elongate the primer up to the length corresponding to synthesis beyond the cross-link site (Figure 4). As yPOLη behaved very similarly, this prominent readthrough appears to be an intrinsic feature of POLη. Previously, hPOLη was reported to be completely blocked in a “standing-start” mode (i.e., when the primed ends immediately before the lesion) by DPC containing a model adduct of green fluorescent protein through 5-(octa-1,7-diynyl)-uracil [36] or H2A or H4 histones conjugated through 5-formylcytosine [39]. However, our system has different cross-linking chemistry and, in particular, places the protein part mostly in the minor DNA groove [42], whereas pyrimidine C5-conjugates face the major groove. Moreover, the primer end in our system is remote from the DPC (Figure 4), facilitating polymerase binding and likely allowing for a more efficient reaction. Structurally, the ability of hPOLη to incorporate a dNMP opposite large and conformationally restrained lesions, such as cyclobutane pyrimidine dimers or platinum cross-links or monoadducts, is partly due to a sharp strand kink at the problematic template site. This movement disengages the template strand 3′ to the lesion from the gap between the finger and the thumb subdomains, relieving the steric conflicts at the bulky lesions [64,65,66,67]. A similar kink, observed in the structure of the Klenow fragment (KF) of *E. coli* DNA polymerase I, facilitates the approach of KF to large protein obstacles tightly bound to DNA, such as Cas9 ribonucleoprotein complex [68]. It is possible that a similar DNA conformation could also be adopted in a DPC and partially relieve the strain caused by the polymerase–DPC clash. It also should be noted that our model DPC is based on the mechanism-based trapping of Fpg DNA glycosylase by NaBH_4_ reduction [42,69,70]. The resulting DPC is linked to DNA through an open abasic (AP) site, and the lack of the coding base may serve as an additional obstacle for the DNA polymerase [71,72,73,74,75,76]. POLη is one of the most effective DNA polymerases to bypass AP sites [75,77], which could contribute to primer elongation beyond the model DPC.

Alternative explanations for apparent primer elongation deep into the DPC are also possible. DNA polymerases (including family Y polymerases) can use a DNA lesion “skipping” mechanism due to Streisinger slippage or dNTP-stabilized misalignment on a template with repetitive bases [76,79,80,81,82,83]. Previously we demonstrated that human PrimPol, an enzyme known to utilize a lesion “skipping” mechanism [84,85], partially bypasses a DPC [45]. The 3′-end primer G expansion following primer dislocation and slippage without direct DPC bypass is also possible.

The situation when a DPC is located in the displaced strand rather than in the template is of a particular interest. Despite some DNA polymerases being well known for their strand displacement activity, the consequences of having a DPC in the displaced strand remain largely unexplored. Strand displacement may occur in many biologically relevant situations, such as repair synthesis, viral DNA replication, etc., and the presence of a DPC could interfere with these processes. In contrast to the situation when a DPC is located in the template, bypassing a DPC in the displaced strand does not involve dNMP insertion opposite a covalent protein adduct. Moreover, the presence of a cross-link could partially melt and unwind the DNA, which may ease strand separation. However, almost all DNA polymerases studied here and in [38], and primase-polymerase PrimPol [45] demonstrated equally efficient or even worse synthesis on the substrates containing a DPC in the displaced strand compared to a DPC in the template strand of a DNA duplex. Even DNA polymerases with strong strand displacement activity could not read through a DPC in the displaced strand. It appears that the bulky protein interferes with parts of the DNA polymerase molecule that make contacts with the displaced strand, thereby preventing the strand from threading through some restricted space. However, the general mechanism of this process remains to be investigated.

Overall, it is clear that POLι, POLη, and POLζ, despite their ability to incorporate dNMPs opposite to a wide variety of lesions, cannot bypass the model DPC, and likely other DPCs as well. The only DNA polymerase that shows this property in vitro, albeit with quite a low efficiency, is PrimPol [45]. These observations suggest that DPCs are unlikely to be tolerated in cells through TLS and that proteolytic processing probably remains the only viable option for dealing with DPCs. However, since POLη and POLζ could extend the primer well into the cross-linked protein’s footprint, up to the cross-link site or even 1–2 nt beyond, it is feasible that these polymerases could strongly distort or even partially unfold the cross-linked protein globule. This might expose normally buried peptides to solution and serve as one of the signals triggering the activity of SPRTN or other DPC repair proteases or ubiquitin ligases targeting the DPC for destruction. An advantage of such a mechanism would be its independence from the nature of the cross-linked protein. We hypothesize that, together with the replicative family B DNA polymerases (POLδ and POLε), POLη and POLζ might participate in the initial stages of DPC repair in human cells.

## Figures and Tables

**Table 1:** Oligonucleotides used in this study.

Sequence, 5′→3′	Length	Function
GCTCTGGAATTCCTTC**X**CTTCTTTCCTCTCGACGGTCTCG X = 8-oxoguanine	40	Template for ss-DPC or temp-DPC, cross-link at X
GCTCTGGAATTCCTTCGCTTCTTTCCTCTCGACGGTCTCG	40	Template for undamaged substrates
GCTCTGGAATTCCTTCCCTTCTTTCCTCTCGACGGTCTCG	40	Template for down-DPC
GAGGAAAGAAG**X**GAAGGAATTCCAGAGC X = 8-oxoguanine	28	Displaced strand for down-DPC, cross-link at X
GAGGAAAGAAGCGAAGGAATTCCAGAGC	28	Displaced strand for undamaged substrates
CGAGACCGTCG	11	Primer and size marker M1
CGAGACCGTCGCGAGGAAAGAAG	23	Size marker M2, corresponding to primer elongation to the cross-link site
CGAGACCGTCGCGAGGAAAGAAGCGAAGGAATTCCAGAGC	40	Size marker M3, corresponding to the full-size product

## Data Availability

All data are contained in the paper.
